# Tissue Localization of Lymphocystis Disease Virus (LCDV) Receptor-27.8 kDa and Its Expression Kinetics Induced by the Viral Infection in Turbot (*Scophthalmus maximus*)

**DOI:** 10.3390/ijms161125974

**Published:** 2015-11-05

**Authors:** Xiuzhen Sheng, Ronghua Wu, Xiaoqian Tang, Jing Xing, Wenbin Zhan

**Affiliations:** 1 Laboratory of Pathology and Immunology of Aquatic Animals, Ocean University of China, 5 Yushan Road, Qingdao 266003, China; xzsheng@ouc.edu.cn (X.S.); starwu061@126.com (R.W.); tangxq@ouc.edu.cn (X.T.); xingjing@ouc.edu.cn (J.X.); 2 Function Laboratory for Marine Fisheries Science and Food Production Processes, Qingdao National Laboratory for Marine Science and Technology, No. 1 Wenhai Road, Aoshanwei Town, Jimo, Qingdao 266071, China

**Keywords:** lymphocystis disease virus, turbot (*Scophthalmus maximus*), receptor-27.8 kDa, expression dynamics, tissue localization

## Abstract

The 27.8 kDa membrane protein expressed in flounder (*Paralichthys olivaceus*) gill cells was proved to be a receptor mediating lymphocystis disease virus (LCDV) infection. In this study, SDS-PAGE and Western blotting demonstrated that 27.8 kDa receptor (27.8R) was shared by flounder and turbot (*Scophthalmus maximus*). Indirect immunofluorescence assay (IIFA) and immunohistochemistry showed that 27.8R was widely expressed in tested tissues of healthy turbot. The indirect enzyme-linked immunosorbent assay indicated that 27.8R expression was relatively higher in stomach, gill, heart, and intestine, followed by skin, head kidney, spleen, blood cells, kidney and liver, and lower in ovary and brain in healthy turbot, and it was significantly up-regulated after LCDV infection. Meanwhile, real-time quantitative PCR demonstrated that LCDV was detected in heart, peripheral blood cells, and head kidney at 3 h post infection (p.i.), and then in other tested tissues at 12 h p.i. LCDV copies increased in a time-dependent manner, and were generally higher in the tissues with higher 27.8R expression. Additionally, IIFA showed that 27.8R and LCDV were detected at 3 h p.i. in some leukocytes. These results suggested that 27.8R also served as a receptor in turbot, and LCDV can infect some leukocytes which might result in LCDV spreading to different tissues in turbot.

## 1. Introduction

Turbot (*Scophthalmus maximus*), a widely cultured marine fish of considerable economic importance in Europe and China, is suffering from various diseases, which cause huge economic losses [1,2,3]. Among them, lymphocystis disease, an infectious viral disease affecting more than 140 marine and freshwater fish species worldwide, which is characterized by formation of papilloma-like lesions on the body surface and sometimes in the internal tissues, rarely causes death in turbot but the diseased fish become more susceptible to secondary infection by other microorganisms, resulting in high mortalities [4,5]. To date, the research on lymphocystis disease in turbot is far from enough. Limited studies mainly use lymphocystis disease virus (LCDV), the causative agent of lymphocystis disease, as a pathogen to explore the molecules involved in the immune response and disease resistance of turbot [6,7,8]. The information concerning the cellular receptors responsible for LCDV infection in turbot is lacking.

Viral receptors are the primary determinants of tissue tropism, which play an important role in the pathogenicity of viruses [9,10,11], and may be shared by different hosts. For LCDV, a 27.8 kDa receptor (27.8R) has been found to be involved in viral attachment and entry in flounder (*Paralichthys olivaceus*) gill cells [12,13]. Since many fish can be infected with LCDV, we suppose that common receptors for LCDV may exist in different fish species, and the 27.8R may be the common receptor shared by flounder and other fish; however, direct evidence is required. Furthermore, in gilthead seabream (*Sparus aurata*), the presence of LCDV in blood samples is verified by PCR [14]. Thus, detection of 27.8R expression and LCDV particles in the blood cells of infected turbot will broaden our understanding of cellular tropism and spreading of LCDV.

The present study attempted to confirm that 27.8R identified from flounder may also serve as a LCDV receptor in turbot, investigate the tissue distribution of 27.8R in healthy turbot using monoclonal antibodies (MAbs) against 27.8R via indirect immunofluorescence assay (IIFA) and immunohistochemistry (IHC), and it also studied the expression variation of 27.8R in response to LCDV infection by indirect enzyme-linked immunosorbent assay (ELISA). The changes of LCDV copy numbers during the infection were detected by real-time quantitative PCR (qPCR). Additionally, the distribution of 27.8R and LCDV in peripheral blood cells of infected turbot was also investigated. The resultant data would promote the understanding of the functional role of 27.8R in pathogenesis and transmission of LCDV in turbot.

## 2. Results

### 2.1. Expression of 27.8R in Turbot by Western Blotting

**Figure 1:**
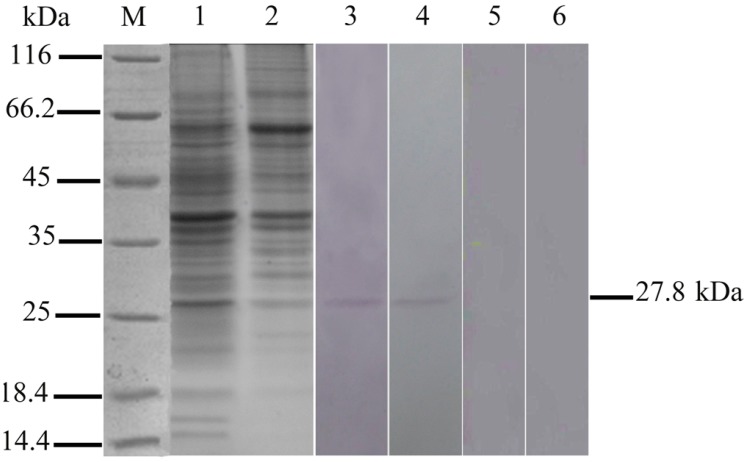
SDS-PAGE and Western blotting showing a band with the molecular size of the 27.8R receptor in turbot gill membrane proteins. M: Molecular mass marker; **Lanes 1** and **2**: SDS-PAGE of flounder and turbot gill membrane proteins, respectively, stained with coomassie blue; **Lanes 3** and **4**: reaction with anti-27.8R MAbs showed only one 27.8 kDa band in flounder and turbot gill membrane proteins; **Lanes 5** and **6**: anti-white spot syndrome virus (WSSV) MAb 1D5 instead of anti-27.8R MAbs served as negative controls.

To confirm 27.8R was shared by flounder and turbot, gill membrane of fish was subjected to SDS-PAGE and Western blotting analysis. Western blotting on gill membrane protein extracted from flounder showed a band of molecular weight of 27.8 kDa (Figure 1, lane 3), which was also observed in the membrane protein of turbot gill (Figure 1, lane 4), indicating that 27.8R was shared by this two fish species. No bands appeared in the negative controls (Figure 1, lanes 5 and 6).

### 2.2. 27.8R Distribution in Turbot by IIFA

**Figure 2:**
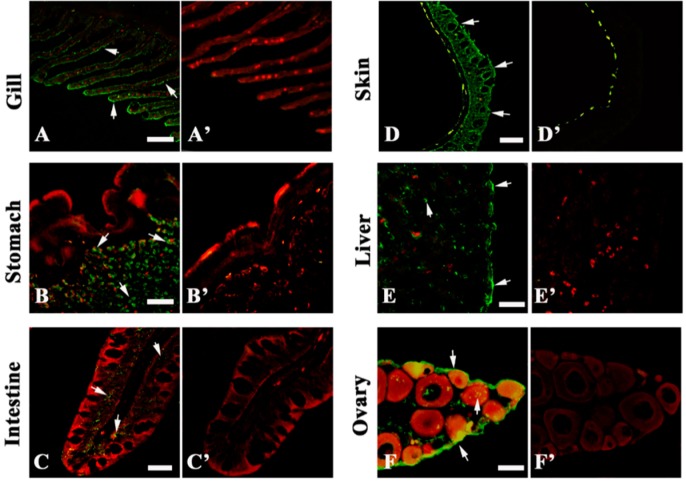
Tissue distribution of 27.8R in turbot detected by indirect immunofluorescence assay (IIFA) under a fluorescence microscope. The green fluorescence (arrow) indicated positive signals of 27.8R. Experimental groups (**A**–**F**): gill (**A**); stomach (**B**); intestine (**C**); skin (**D**); liver (**E**); and ovary (**F**) were incubated with anti-27.8R Mabs. Negative control groups (**A’**–**F’**) were incubated with anti-WSSV MAb 1D5. All tissues were stained in red by EBD. Bar = 50 μm.

IIFA using MAbs against 27.8R was carried out to detect tissue distribution of 27.8R in turbot. Evident green fluorescence was observed in the stomach gland, gill epithelial cells, hepatocytes, ovary, intestinal mucosal epithelium and skin epithelial layer (Figure 2), the positive signals also distributed in head kidney, spleen cells, tubular epithelium of kidney, cardiac cells, and the pallium of healthy turbot (Figure S1), indicating the distribution of 27.8R in these tissues. No green fluorescence was observed in the negative controls except in skin melanin where auto-fluorescence might occur (Figure 2D,D’). All tested tissues, including negative controls, were non-specifically stained by Evan’s blue dye (EBD) and fluoresced red under a fluorescence microscope.

### 2.3. Localization of 27.8R by Immunohistochemistry

**Figure 3:**
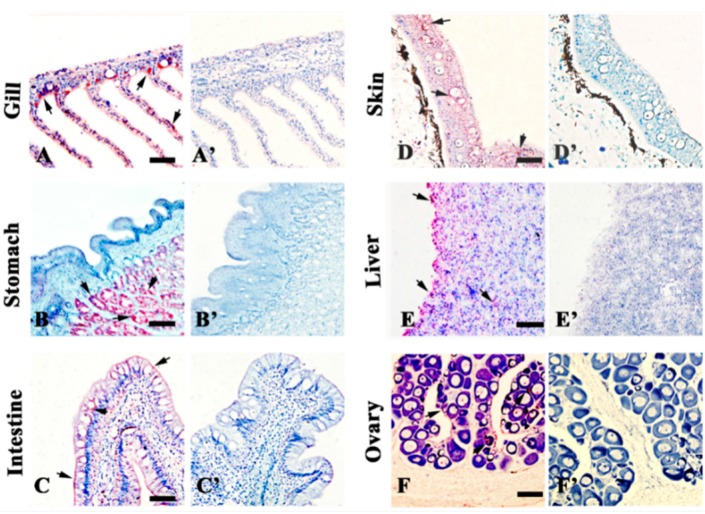
Immunohistochemical detection of 27.8R in tissues of turbot. Anti-27.8R Mabs positive signals (arrow) were observed in gill (**A**); stomach (**B**); intestine (**C**); skin (**D**); liver (**E**); and ovary (**F**), showing the distribution of 27.8R. No red signal was present in negative controls (**A’**–**F’**), which used anti-WSSV MAb 1D5 as primary antibody. All tissues were counterstained with Mayer’s hematoxylin. Bar = 50 μm.

To confirm the reliability of the IIFA results, immunochemical detection using anti-27.8R Mabs was performed on tissues of healthy turbot described above. Consistent with the results of IIFA, positive red signals were observed in the stomach gland epithelium, gill epithelial cells, intestinal mucosal epithelium, epidermis of skin, hepatocytes, and ovary (Figure 3), the positive signals also distributed in cardiac cells, head kidney, spleen cells, tubular epithelium of kidney, and the pallium (Figure S2), showing the existence of 27.8R. No red positive signal was observed in the negative controls.

### 2.4. Expression Changes of 27.8R after LCDV Infection

**Figure 4:**
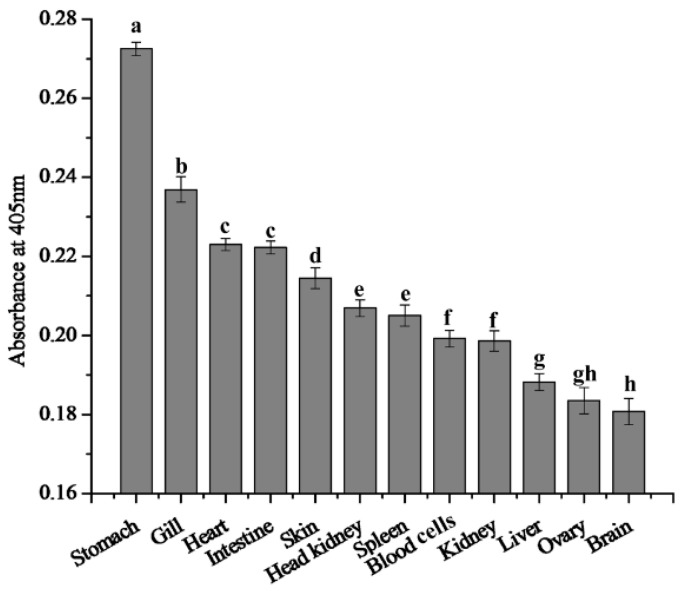
Comparison of 27.8R expression in tissues of turbot at 0 h p.i. Error bars represented S.D., data represented the absorbance value at 405 nm (mean ± S.D.; *n* = 3). Data were compared by one-way ANOVA. Means with different letters were significantly different (*p* < 0.05).

The changes of 27.8R expression in tissues post LCDV infection was monitored via ELISA, and the expression of 27.8R was determined by absorbance value at 405 nm. In the healthy turbot, the expression of 27.8R was relatively higher in the stomach, gill, heart, and intestine, followed by skin, head kidney, spleen, blood cells, kidney and liver, and lower in ovary and brain (Figure 4).

**Figure 5:**
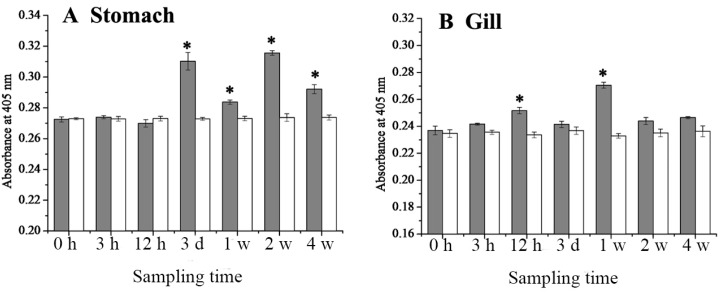

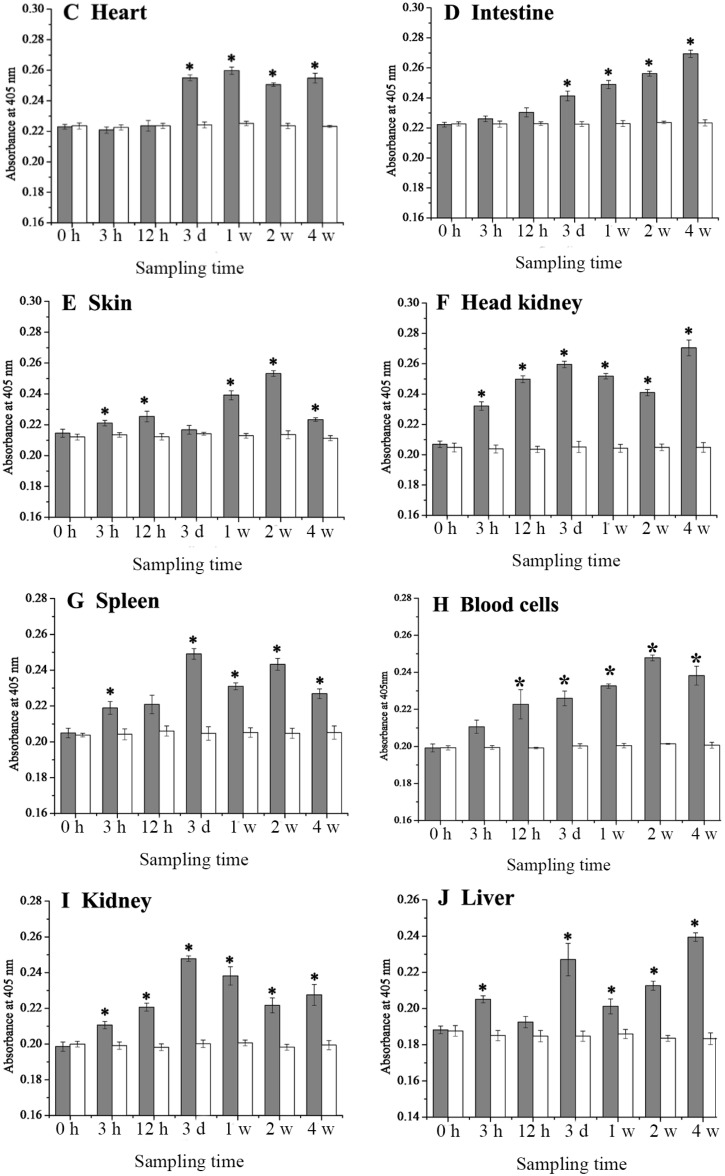

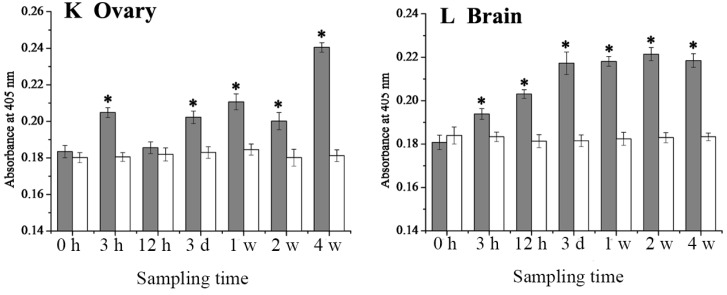
The expression dynamics of 27.8R in tissues of turbot determined by ELISA., including (**A**) stomach; (**B**) gill; (**C**) heart; (**D**) intestine; (**E**) skin; (**F**) head kidney; (**G**) spleen; (**H**) blood cells; (**I**) kidney; (**J**) liver; (**K**) ovary; (**L**) brain. Error bars represented S.D., data represented the absorbance value at 405 nm (mean ± S.D.; *n* = 3). Grey column and white column represented the expression of 27.8R in tissues of turbot after challenged with LCDV and sterile PBS (control), respectively. Data were compared by Student’s *t* test. The asterisk represented the statistical significance (*p* < 0.05) as compared with the control. h: hours; d: days; w: weeks.

After challenged by virus infection, the expression of 27.8R in tissues and blood cells was significantly up-regulated (*p* < 0.05), and kept a relatively higher level in the stomach (Figure 5A), heart (Figure 5C), intestine (Figure 5D), and head kidney (Figure 5F), but a relatively lower level in ovary (Figure 5K) and brain (Figure 5L). In the control group challenged with sterile phosphate buffered saline (PBS, pH 7.4), expression of 27.8R showed no significant difference over four weeks (Figure 5).

### 2.5. Dynamics of LCDV Copies in Fish Tissues

**Figure 6:**
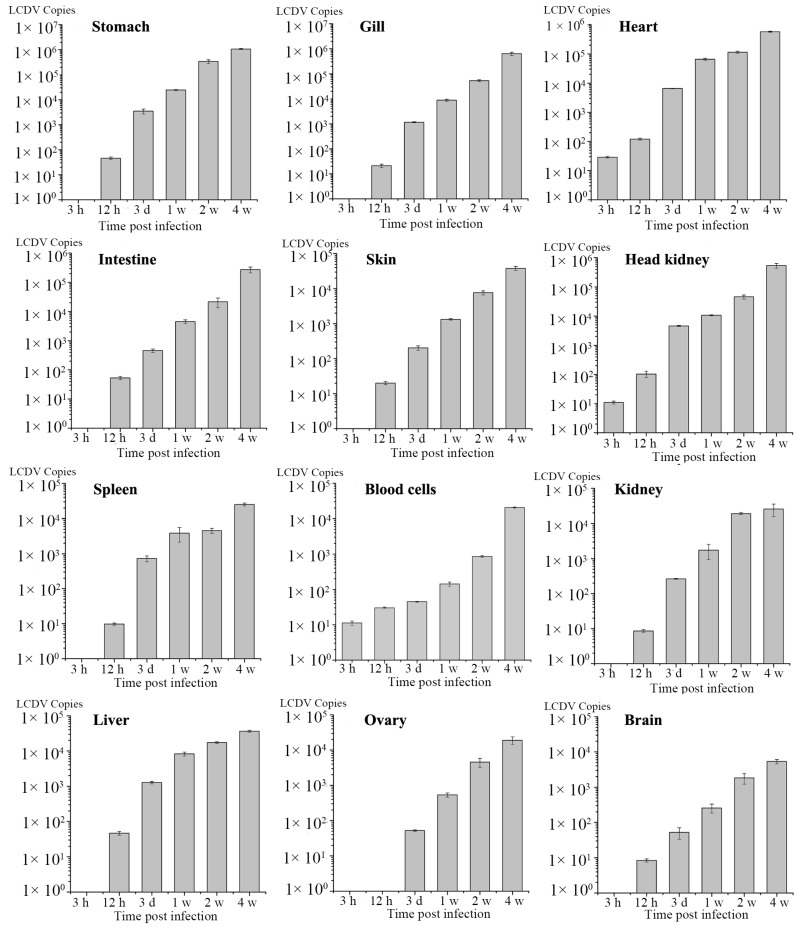
Dynamics of LCDV replication in tissues during four weeks of LCDV infection investigated by qPCR. Error bars represented S.D., data represented the number of LCDV copies per microgram of total DNA in tissue samples (mean ± S.D.; *n* = 3). h: hours; d: days; w: weeks.

**Figure 7:**
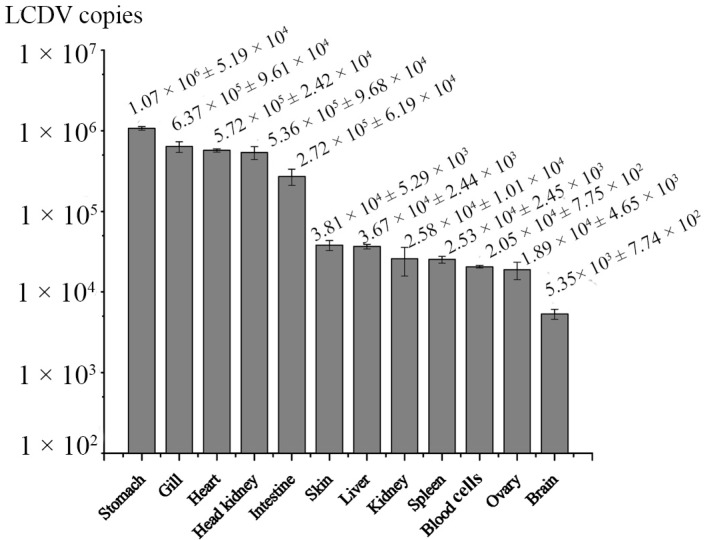
Comparison of LCDV loads among tissues at 4 w p.i. investigated by qPCR. Error bars represented S.D., data represented the number of LCDV copies per microgram of total DNA in tissue samples (mean ± S.D.; *n* = 3).

The LCDV copies in tissues at different time point post infection was determined by absolute qPCR. During four weeks of infection with LCDV, no clinical symptoms were observed in turbot. LCDV copy numbers per microgram of total DNA in tissue samples were calculated by extrapolating values from the standard curve. LCDV was firstly detected in heart, head kidney and blood cells at 3 h p.i., and then in other tested tissues at 12 h p.i., with exception of ovary where LCDV copies were not detectable until 3 d (days) p.i. (Figure 6). LCDV copies increased in a time-dependent manner in all tested tissues, and reached the maximum value at 4 w (weeks) p.i., which was highest in stomach (1.07 × 10^6^), followed by gill, heart, head kidney, and intestine (7 × 10^5^~2 × 10^5^), and then in skin, liver, kidney, spleen, blood cells, and ovary (4 × 10^4^~1 × 10^4^), and lowest in the brain (5.35 × 10^3^) (Figure 7).

### 2.6. 27.8R Distribution and LCDV Antigens in Peripheral Blood Cells

**Figure 8:**
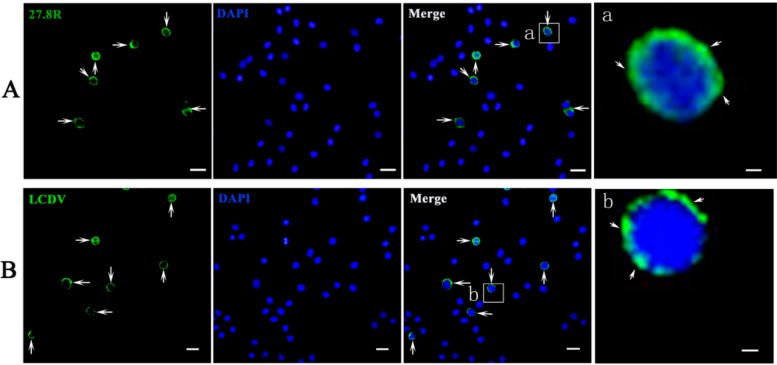
Detection of 27.8R expression and LCDV particles in peripheral blood cells by IIFA. Peripheral blood cells were isolated from turbots at 3 h p.i. and stained with anti-27.8R MAbs and anti-LCDV MAb for detection of 27.8R and LCDV, respectively. The green fluorescence (arrow) indicated the positive signals of 27.8R (**A**) or LCDV (**B**). Cell nuclei were counterstained in blue by DAPI. Scale bar = 10 μm. (**a**,**b**) were the higher magnification view of the insert area in the merge pictures showing 27.8R and the LCDV positive area, respectively. Scale bar = 1 μm.

MAbs against 27.8R and LCDV were used to detect the 27.8R distribution and LCDV antigens, respectively, in red blood cell and whole blood cell smears of turbot at 3 h post LCDV infection, respectively. In red blood cells, no green fluorescence signals were observed for 27.8R or LCDV (Figure S3), indicating that 27.8R expression, as well as LCDV binding, do not occur in these cells. For detection of 27.8R distribution in a whole blood cell smear, the green fluorescence mainly distributed in the membrane surface of a small portion of blood cells, indicating the existence of 27.8R (Figure 8A). For LCDV detection, green signals were mainly present at the surface of a small portion of blood cells (Figure 8B). In the negative control, no green fluorescence distributed in cells stained by anti-WSSV MAb 1D5 (Figure S4). DAPI nuclear staining is shown in blue.

## 3. Discussion

The first stage of viral entry is the absorption or attachment onto the cells holding a receptor that the virus could bind to, followed by internalization and uncoating [15], and investigation upon expression changes of the cellular receptor during virus infection will contribute to clarifying the pathogenesis of viral infection. In the previous research, 27.8R was identified and found to be responsible for LCDV attachment and entry in flounder gill cells, and anti-27.8R MAbs were developed [12,13]. In this study, to test whether the 27.8R is a common receptor shared by flounder and turbot, Western blotting analysis of turbot gill membrane proteins was performed, showing one band with a molecular weight of 27.8 kDa; moreover, 27.8R was found to distribute widely in the tested tissues of turbot, and obvious up-regulation of its expression was observed after LCDV infection. These results suggested that 27.8R identified in flounder also served as a cellular receptor for LCDV in turbot. Additionally, the widespread distribution of 27.8R suggested a broad range tissue tropism of LCDV in turbot, and this was verified by qPCR results which showed LCDV genome copy numbers increased in a time-dependent manner in the tested tissues of turbot, indicating LCDV could replicate in these target tissues. So far, LCDV or lymphocystis cells have been detected in tissues including gill, stomach, intestine, skin, heart, brain, liver, spleen, head kidney, kidney, and gonad in different fish [16,17,18,19,20,21], which is consistent with our results. However, whether the 27.8R is expressed in these tissues of other fish species and serves as a receptor is worth further research.

Virus replication was more efficient when target cells over-expressed the receptor [22,23,24,25]. In this study, significant up-regulation of 27.8R expression in response to LCDV challenge was observed. Interestingly, high LCDV load generally occurred in the tissues where the expression of 27.8R was relatively higher before and after LCDV infection, such as stomach, gill, heart and intestine, providing the possibility of a positive correlation that higher 27.8R expression would facilitate a more efficient LCDV proliferation. Similar correlation was previously found in flounder gill cells [26]. Therefore, LCDV infection could contribute to an up-regulation of 27.8R expression in turbot which, in turn, enhanced the sensitivity of fish tissues to viral infection, resulting in efficient LCDV replication. In addition, formation of papilloma-like lesions in the skin is one of the most obvious symptoms of lymphocystis disease in many fish, including flounder and gilthead seabream [5,14]. However, in turbot, expression of 27.8R in skin was relatively low, and similar results were also observed in flounder skin [27], so we deduced that other cellular receptor besides 27.8R might exist in skin, such as 37.6 kDa receptor protein [28]. While the LCDV genome copy number was also low in skin at 4 weeks p.i., therefore, further studies are needed.

The references have shown that the main mode of fish-to-fish transmission of LCDV is horizontally via direct contact and external trauma [29,30], and skin and gill were the main ports of entry [31,32]. Although inoculation by LCDV injection cannot reflect the natural infection mode, it can ensure that each fish is inoculated with the same dose of LCDV, so viral replication and LCDV-induced 27.8R expression were investigated after LCDV injection in the present study. It was found that viral copies were firstly detected in heart, blood cells, and head kidney at 3 h after intramuscular injection with LCDV, and then detected in other tested tissues from 12 h post infection, which indicated that a systemic infection was probably caused by virion entry into the bloodstream, as proposed by Colorni *et al.* [17] and confirmed in gilthead seabream [14,21,33]. Furthermore, 27.8R distribution and LCDV antigens were detected in a small portion of peripheral blood cells but red blood cells were negative for LCDV presence, revealing that LCDV could not infect the red blood cells but could infect some kinds of leukocytes. These leukocytes might play important roles in LCDV spreading between tissues in turbot. Further studies to characterize the specific subset of the leukocytes which are susceptible to LCDV are needed. Additionally, the kinetics of LCDV replication and 27.8R expression in turbot after bath exposure to LCDV are worthy of further research.

## 4. Materials and Methods

### 4.1. Ethics Statement

This study was carried out strictly in line with the procedures in the Guide for the Use of Experimental Animals of the Ocean University of China. In this study, for the methods used in animal experiments were approved by the Institutional Animal Care and Use Committee of Ocean University of China (Permit Number: 20111201). All efforts were dedicated to minimize suffering. The NC3Rs ARRIVE Guidelines checklist was shown in the Checklist S1 [34].

### 4.2. Fish, Virus and Monoclonal Antibodies

Healthy turbots, weighing 700–750 g, were obtained from a local fish farm in Qingdao, Shandong province, China, and confirmed to be LCDV-free by nest PCR [16]. The fish were kept in rearing tanks supplied with running aerated seawater at 18 ± 1 °C and fed daily with dry food pellets for one week before LCDV exposure.

Four flounders infected with lymphocystis disease, featured by lymphocystis nodules on the skin, fin and gill, were donated by a fishing farm in Qingdao, Shandong province of China. The isolation and purification of LCDV particles were conducted in strict accordance with the methods used in Cheng *et al.* [35]. After purification, virus was suspended in sterile 0.01 M PBS and stored at −80 °C, and the concentration of LCDV was determined [12].

The MAbs against 27.8R (3D9:2G11 = 1:1, *v*/*v*) [13], Mab 1A8 against LCDV [35], as well as MAb 1D5 against WSSV [36] were previously produced by our lab.

### 4.3. SDS-PAGE and Western Blotting Analysis

SDS-PAGE and Western blotting of gill membrane protein using MAbs against 27.8R were performed. Gill membrane protein from flounder served as a positive control. The membrane proteins were extracted according to Wang *et al.* [12], gill tissues from healthy turbot and flounder were homogenized in lysis buffer (137 mM NaCl, 250 mM sucrose, 10% glycerol and 1% NP-40, 20 mM Tris–HCl, pH 8.0) containing the protease inhibitor cocktail (Roche, Mannheim, Germany). Then the tissue homogenate was subjected to differential centrifugations to get membrane proteins.

Gill membrane proteins were suspended and adjusted to a concentration of 1 mg/mL in PBS. For Western blotting analysis, the membrane proteins were separated by SDS-PAGE and transferred onto polyvinyldifluoride (PVDF) membrane (Millipore, Bedford, MA, USA). After blocking with 3% bovine serum albumin (BSA, Sigma, Saint Louis, MO, USA) in sterile PBS for 1 h at 37 °C, the PVDF membrane was incubated with MAbs against 27.8R (1:1000) for 1 h at 37 °C. After washing three times with PBST (PBS containing 0.05% Tween-20), and alkaline phosphatase (AP)-conjugated goat-anti-mouse Ig (1:3000, Sigma) was added for 1 h incubation at 37 °C. Finally, positive bands were developed with substrate solution containing nitroblue tetrazolium chloride (NBT, Sigma) and 5-bromo-4-chloro-3-indolylphosphate toluidine salt (BCIP, Sigma) for 5 min, the reaction was stopped by washing with distilled water. As a negative control, incubation with MAb 1D5 against WSSV (1:1000), instead of MAbs against 27.8R, was conducted.

### 4.4. Tissue Cryosections and IIFA

A total of 11 fish tissues including gill, stomach, intestine, spleen, ovary, skin, liver, head kidney, kidney, brain, and heart were collected from three healthy individuals. Tissue cryosections were prepared according to Lin *et al.* [37]. For IIFA, sections were incubated with MAbs against 27.8R (1:1000) and FITC-conjugated goat anti-mouse IgG (1:256, Sigma) containing 1 μg/mL EBD (Fluka, Lyon, France) as the counterstain. Incubation with MAb 1D5 against WSSV (1:1000), instead of MAbs against 27.8R, acted as a negative control. All incubations were conducted at 37 °C for 1 h in a moisture chamber in the dark, and slides were washed three times with PBS after incubation. The slides were rinsed again and observed under a fluorescence microscope.

### 4.5. Tissue Paraffin Sections and Immunohistochemistry

The same tissues as mentioned above were fixed with Bouin’s fixative and then rinsed with 70% alcohol. Subsequently, tissues were dehydrated and embedded in paraffin wax, followed by preparation of 7-μm sections. After deparaffination in xylene and rehydration in ethanol series, the antigen retrieval protocol was carried out as described by Faulk *et al.* [38]. Briefly, a rack of sections were totally immersed in sodium citrate buffer (10 mM, pH 6.0), heated to 95 °C for 20 min and then cooled to room temperature. The slides were rinsed in 0.5 M EDTA for 1 h at room temperature to inhibit endogenous AP, pre-incubated with 3% (*w*/*v*) BSA in PBS for 1 h to block non-specific antibody binding, and followed by an incubation with anti-27.8R Mabs (1:1000) at 37 °C for 1 h in a moisture chamber. MAb 1D5 against WSSV (1:1000) replaced Mabs against 27.8R as the negative control. After washing three times with PBS, the sections were incubated with biotinylated horse anti-mouse IgG (H + L, heavy and light chain) and streptavidin-alkaline phosphatase (S-A/AP) at a dilution ratio of 1:300 in PBS for 1 h at 37 °C. After three washes in PBS, immunodetection was performed by incubating the slides with AP-Red substrate kit and counterstained with Mayer’s hematoxylin for 10 min, and then examined under a light microscope (Olympus, Monolith, Japan).

### 4.6. Virus Infection and Sampling

For virus infection assay, the fish were randomly divided into two groups, thirty fish of group one was injected intramuscularly with 300 μL purified LCDV (100 μg per fish) as experimental group, and thirty fish of group two was injected intramuscularly with equal volume of sterile PBS as control. The 11 tissues mentioned above, and peripheral blood cells were sampled from three individuals at 0, 3 and 12 h, and 3 days, 1 week, 2 weeks and four weeks post infection (p.i.), tissue samples were split for both ELISA and qPCR assay. In addition, peripheral blood cells were separated from turbots at 3 h post LCDV intramuscular injection, and then the blood cell smears were prepared for detection of 27.8R and LCDV distribution.

### 4.7. ELISA

For detecting the expression changes of 27.8R during LCDV infection, the membrane proteins of each sampled tissue and peripheral blood cells were extracted as up-mentioned and adjusted to a concentration of 100 μg/mL, followed by coating with 100 μL of membrane proteins in triplicate in 96-well plates (Costar) for 12 h at 4 °C per well. Subsequently, the wells were washed thrice with PBS and blocked with 3% BSA in 200 μL PBS for 1 h at 37 °C. After washing thrice with PBS, each well was incubated with 100 μL of Mabs against 27.8R for 1 h at 37 °C. MAb 1D5 against WSSV replaced MAbs againt 27.8R as the negative control. Following three washes, 100 μL of goat anti-mouse Ig-alkaline phosphatase conjugate (Sigma) diluted 1:1000 in PBS was added and incubated for 1 h at 37 °C. After the last washing, 100 μL of 0.1% (*w*/*v*) pNPP (Sigma) carbonate-bicarbonate buffer containing 0.5 mM MgCl_2_ was added to each well and incubated for 30 min at room temperature in the dark. The reaction was stopped with 50 μL per well of 2 M NaOH and absorbance values were measured at 405 nm with an automatic ELISA reader (Tecan, Männedorf, Switzerland).

### 4.8. Real-Time Quantitative PCR

For LCDV quantification by qPCR, TIANamp Marine Animals DNA Kit was used to extract total DNA of the sampled tissues and peripheral blood cells. A qPCR protocol in line with Zhu *et al.* [39] with some modifications was developed. Copy numbers of LCDV major capsid protein (MCP) gene was quantified according to a standard curve method. Briefly, PCR primer sets P1/P2 designed by Zhan *et al.* [16] were used to amplify *MCP* gene (348-bp) of LCDV. Subsequently, positive control plasmid, which contained the *MCP* gene, was constructed and purified. The positive plasmid was subjected to sequencing to ensure the presence of the target sequence. Then, spectrophotometric analysis was carried out to determine the concentration of the positive plasmid DNA, and the gene copy number was determined according to the molar mass derived from the plasmid DNA containing the 348-bp insert. Serially ten-fold diluted plasmids, from 1.9 × 10^1^ to 1.9 × 10^9^ copies, were prepared as standard samples. Finally, the primer sets P3/P4 designed by Zhan *et al.* [16], which targeted a partial sequence of the MCP gene (173-bp), was used in the qPCR reaction. The samples were subjected to PCR reaction alongside the serially-diluted plasmid DNA standard and run in four replicates. After reaction, the Ct value for each PCR sample was determined automatically by the software accompanying the PCR system (Roche Light Cycler^®^ 480 system), and the curve for standard dilutions was calculated based on the Ct values. The LCDV genome copy numbers for the DNA samples were determined by extrapolating values from the standard curve.

### 4.9. Blood Cell Smear Preparation and IIFA

The 27.8R localization and LCDV antigens were detected in peripheral blood cells of turbot at 3 h p.i. Blood samples were averagely divided into two groups, group one was directly diluted in PBS to 10^6^ cells/mL, and group two was centrifuged at 100× *g* for 20 min at 4 °C and the red blood cell pellet was re-suspended and diluted in PBS to 10^6^ cells/mL. The whole blood cells and red blood cells were then settled on glass slides for 2 h, and fixed for 20 min at 22 °C with 4% paraformaldehyde. For immune detection of 27.8R localization and LCDV antigens, both blood cell smears were incubated with MAbs against 27.8R (1:1000) and MAb 1A8 against LCDV (1:1000), respectively. Incubating with MAb 1D5 against WSSV, instead of anti-27.8R MAbs and anti-LCDV MAb 1A8, acted as the negative control. After washing three times with PBS, the slides were incubated with FITC-conjugated goat-anti-mouse Ig (1:256, Sigma, St. Louis, MO, USA) for 1 h at 37 °C in the dark. DAPI nuclear staining is shown in blue. After three washes with PBS, slides were mounted with 90% glycerin before observation under a fluorescence microscope.

### 4.10. Statistics

All data were expressed as mean ± standard deviation (S.D.). The statistical analysis of 27.8R expression in different tissues of healthy was conducted by one-way ANOVA, and analysis of 27.8R expression in LCDV-infected turbot was carried out by Student’s *t* test using SPSS 17.0 software. Significant differences were considered when *p* < 0.05.

## 5. Conclusions

This study investigated the tissue distribution of 27.8R in healthy turbot, and dynamics of 27.8R expression during LCDV proliferation. It was found that 27.8R was shared by flounder and turbot, and widely distributed in the tested tissues of healthy turbot and its expression was up-regulated in response to LCDV infection. High 27.8R expression had a positive correlation with efficient LCDV proliferation. Moreover, some kinds of leukocytes might play important roles in LCDV transmission between tissues in fish. To our knowledge, this is the first study about the 27.8R contribution to LCDV infection and LCDV spreading in turbot tissues.

## Supplementary Materials

Supplementary materials can be found at http://www.mdpi.com/1422-0067/16/11/25974/s1.
