# Supplementary material for: Tissue Localization of Lymphocystis Disease Virus (LCDV) Receptor-27.8 kDa and Its Expression Kinetics Induced by the Viral Infection in Turbot (Scophthalmus maximus)
Source: Int J Mol Sci. 2015 Nov 5;16(11):26506–19. doi: 10.3390/ijms161125974 (PMC4661833; doi:10.3390/ijms161125974)
Supplement: Supplementary file 1 [file ijms-16-25974-s001.pdf]

International Journal of
Molecular Sciences

MDPI

Supplementary Information

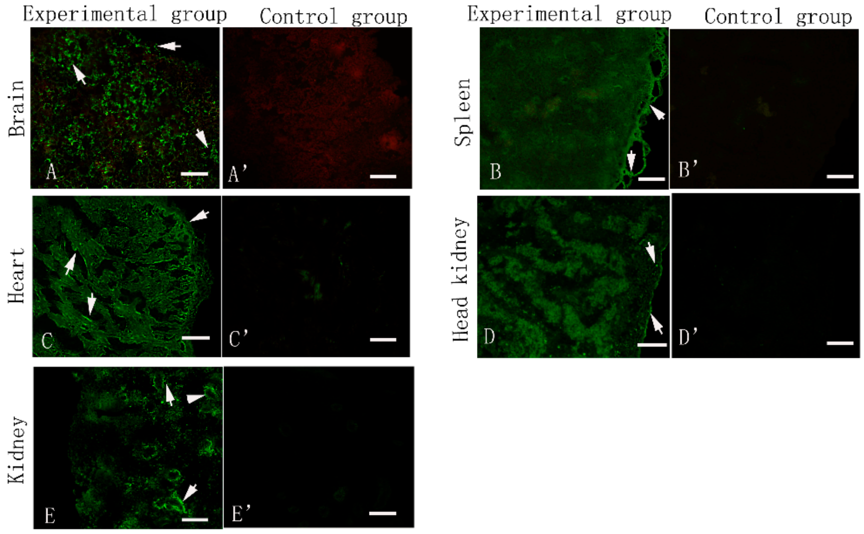

Figure S1. Distribution of 27.8R in (**A**,**A′**) brain; (**B**,**B′**) spleen; (**C**,**C′**) heart; (**D**,**D′**) head kidney and (**E**,**E′**)kidney detected by IIFA.The green fluorescence (white arrow) indicated positive signals of 27.8R. Scale bar: 50 μm.

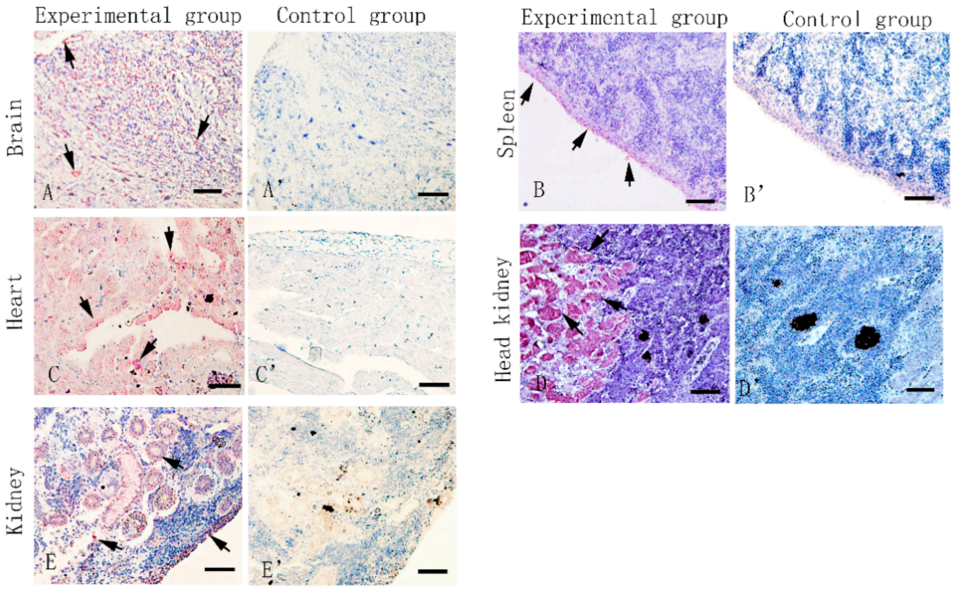

Figure S2. Distribution of 27.8R in (**A**,**A′**) brain; (**B**,**B′**) spleen; (**C**,**C′**) heart; (**D**,**D′**) head kidney and (**E**,**E′**) kidney detected by IHC. Black arrows indicated the distribution of 27.8R. Scale bar: 50 μm.

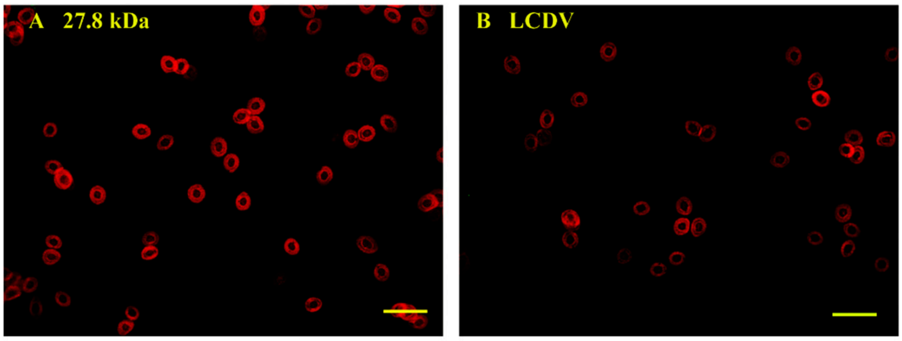

Figure S3. IIFA detection of the (**A**) 27.8-kDa receptor and (**B**) LCDV particles in turbot red blood cells post-LCDV infection, and no green fluorescence was observed. Cells were non-specificly stained in red by EBD. Scale bar: 10 μm.

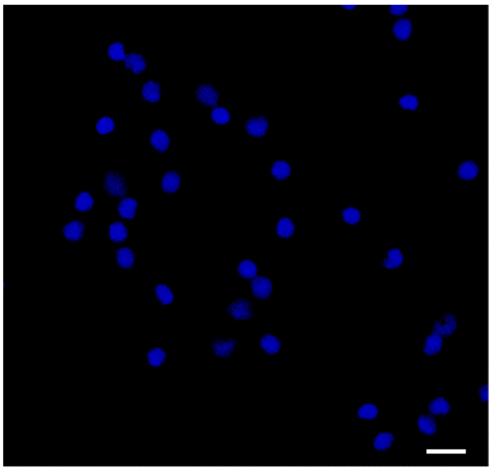

Figure S4. IIFA results of negative control using anti-WSSV MAbs as primary antibody in turbot whole blood cells post-LCDV infection, showing no green fluorescence in the cells. Cell nuclei were counterstained in blue by DAPI. Scale bar: 10 μm.
